# Creatine Enhances the Effects of Cluster-Set Resistance Training on Lower-Limb Body Composition and Strength in Resistance-Trained Men: A Pilot Study

**DOI:** 10.3390/nu13072303

**Published:** 2021-07-04

**Authors:** Diego A. Bonilla, Richard B. Kreider, Jorge L. Petro, Ramón Romance, Manuel García-Sillero, Javier Benítez-Porres, Salvador Vargas-Molina

**Affiliations:** 1 Research Division, Dynamical Business & Science Society–DBSS International SAS, Bogotá 110861, Colombia; jlpetros@hotmail.com; 2 Research Group in Biochemistry and Molecular Biology, Universidad Distrital Francisco José de Caldas, Bogotá 110311, Colombia; 3 Research Group in Physical Activity, Sports and Health Sciences (GICAFS), Universidad de Córdoba, Montería 230002, Colombia; 4 kDNA Genomics®, Joxe Mari Korta Research Center, University of the Basque Country UPV/EHU, 20018 Donostia-San Sebastián, Spain; 5 Exercise & Sport Nutrition Lab, Human Clinical Research Facility, Texas A&M University, College Station, TX 77843, USA; rbkreider@tamu.edu; 6 Body Composition and Biodynamic Laboratory, Faculty of Education Sciences, University of Málaga, 29071 Málaga, Spain; arromance@uma.es; 7 Faculty of Sport Sciences, EADE-University of Wales Trinity Saint David, 29018 Málaga, Spain; manugarciasillero@gmail.com (M.G.-S.); salvadorvargasmolina@gmail.com (S.V.-M.); 8 Physical Education and Sports, Faculty of Medicine, University of Málaga, 29071 Málaga, Spain; benitez@uma.es

**Keywords:** phosphocreatine, muscle fatigue, adipose tissue, muscle strength, dietary supplements, physiological adaptation

## Abstract

Creatine monohydrate (CrM) supplementation has been shown to improve body composition and muscle strength when combined with resistance training (RT); however, no study has evaluated the combination of this nutritional strategy with cluster-set resistance training (CS-RT). The purpose of this pilot study was to evaluate the effects of CrM supplementation during a high-protein diet and a CS-RT program on lower-limb fat-free mass (LL-FFM) and muscular strength. Twenty-three resistance-trained men (>2 years of training experience, 26.6 ± 8.1 years, 176.3 ± 6.8 cm, 75.6 ± 8.9 kg) participated in this study. Subjects were randomly allocated to a CS-RT+CrM (*n* = 8), a CS-RT (*n* = 8), or a control group (*n* = 7). The CS-RT+CrM group followed a CrM supplementation protocol with 0.1 g·kg^−1^·day^−1^ over eight weeks. Two sessions per week of lower-limb CS-RT were performed. LL-FFM corrected for fat-free adipose tissue (dual-energy X-ray absorptiometry) and muscle strength (back squat 1 repetition maximum (SQ-1RM) and countermovement jump (CMJ)) were measured pre- and post-intervention. Significant improvements were found in whole-body fat mass, fat percentage, LL-fat mass, LL-FFM, and SQ-1RM in the CS-RT+CrM and CS-RT groups; however, larger effect sizes were obtained in the CS-RT+CrM group regarding whole body FFM (0.64 versus 0.16), lower-limb FFM (0.62 versus 0.18), and SQ-1RM (1.23 versus 0.75) when compared to the CS-RT group. CMJ showed a significant improvement in the CS-RT+CrM group with no significant changes in CS-RT or control groups. No significant differences were found between groups. Eight weeks of CrM supplementation plus a high-protein diet during a CS-RT program has a higher clinical meaningfulness on lower-limb body composition and strength-related variables in trained males than CS-RT alone. Further research might study the potential health and therapeutic effects of this nutrition and exercise strategy.

## 1. Introduction

Several changes with regard to the synthesis and hydrolysis of adenosine triphosphate (ATP) occur inside muscle cells during all-out short-term physical exercise [1]. This relationship between energy production and consumption (myocellular ATP/ADP ratio) is crucial for the onset of muscle fatigue, which is characterized by an acute reduction in force and power in response to contractile activity [2]. In parallel to the decrease of the ATP/ADP ratio, muscle concentrations of inorganic phosphate (Pi) and hydrogen ions (H^+^) significantly rise over the course of high-intensity physical exercise. This metabolic stress is also currently considered one of the main mechanisms evoking muscle fatigue [3]. Affecting mainly the muscle fibers expressing myosin heavy chain isoform II (fast twitch or type II), the intracellular accumulations of Pi and H^+^ impair the function of the contractile machinery via several mechanisms, including inhibition of actomyosin and sarco/endoplasmic reticulum Ca^2+^-ATPases (SERCA), reduction of the Gibbs free energy of ATP hydrolysis, alteration of the state of actin–myosin cross-bridges, and uncoupling of dihydropyridine and ryanodine receptors [3,4,5]. In fact, it has been proposed that initial phase of force reduction is accompanied by an increase in Pi concentration and dysregulation of Ca^2+^ handling (i.e., disruptions in the Ca^2+^ release–reuptake process in the sarcoplasmic reticulum), suggesting a possible precipitation of calcium phosphate in the sarcoplasmic reticulum [6]. 

It is well known that the metabolism of phosphagen compounds (high-energy phosphates), such as phosphocreatine (PCr), provides an immediate and predominant energy source for the rapid and localized regeneration of ATP during explosive short-duration physical exercise [7]. Interestingly, during periods of muscle inactivity (rest between sets), PCr resynthesis requires energy from ATP hydrolysis to allow the transphosphorylation of free creatine (Cr) by different creatine kinase (CK) isoforms [8]. In comparison to ATP and ADP, PCr and Cr are smaller and less negatively charged molecules that can be found in much higher concentrations in the myocyte, allowing greater intracellular flux through the mitochondrial reticulum [9] and CK/PCr system [10,11]. Hence, the CK/PCr system might be considered as a crucial spatio-temporal energy and metabolic buffer during high-intensity short-duration physical exercise [8]. Optimization of the CK/PCr system can be attained by supplementation with creatine monohydrate (CrM), which is not only the most studied, safe, and effective nutritional ergogenic aid [12,13,14,15] but also has several potential health/therapeutic benefits [16,17,18,19,20,21].

As a type of explosive strength training, progressive heavy resistance training (RT) is recognized by promoting positive musculoskeletal and functional adaptations in a wide range of populations [22,23,24,25]. Traditional set configurations of RT usually involve a number of repetitions performed in a continuous fashion without any pause in between. However, several configurations of cluster-set RT (CS-RT), which includes intra-set rest periods, have been studied with interest in the recent years [26,27,28,29]. This strategy has been reported as a time-efficient tool to attenuate the loss in mean propulsive velocity, power, and peak force [30,31], and to improve the exercise adaptations in strength-trained individuals [32,33]. 

The intra-set rest periods of CS-RT might hypothetically lead to a greater PCr resynthesis, which in turn could optimize the energy and metabolic buffering role of the CK/PCr system. Indeed, it has been proposed that this would control intramuscular pH, avoid accumulation of metabolic products, restore membrane potential to resting values, and increase blood flow reperfusion into the muscle and consequently increase oxygen transport to the tissues [34]. Previous work has shown that during rest PCr resynthesis has a biphasic time course behavior (fast phase during the first 21–22 s and slow phase from 170 s and beyond) [35]. Although different studies have assessed the effects of CS-RT with different configurations on both progressive overload [36,37] and plyometric training [38,39], to our knowledge there is no study that has evaluated the combination of this strategy with CrM supplementation on body composition and strength. Thus, the aim of this pilot study was to evaluate the effects of chronic CrM supplementation (0.1 g·kg^−1^·day^−1^) plus a high-protein diet during an eight-week CS-RT program on lower-limb body composition and strength-related variables in resistance-trained men. We hypothesize that the combination of CrM, a high-protein diet, and CS-RT will have a great impact on exercise adaptations in advanced exercisers. 

## 2. Materials and Methods

### 2.1. Trial Design

**Figure 1:**
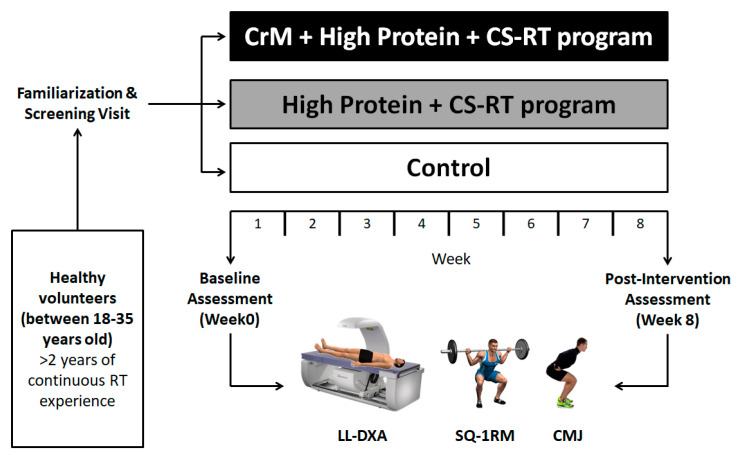
Experimental design of the study. CMJ: countermovement jump; CrM: creatine monohydrate; CS-RT: cluster-set resistance training; LL-DXA: lower-limb assessment with dual-energy X-ray absorptiometry; SQ-1RM: 1 repetition maximum for the back squat.

This was a triple-arm single-blinded and repeated-measures randomized controlled pilot study in resistance-trained men. The study was designed following the Consolidated Standards of Reporting Trials (CONSORT) extension to pilot and feasibility trials with suitable adaptations [40]. We evaluated the effects of CrM supplementation on a high-protein diet during eight weeks of a CS-RT program on lower-limb body composition and strength-related parameters. All variables were measured at baseline (Week 0) and after the CS-RT program (Week 8) (Figure 1).

### 2.2. Participants

A total of 27 men (26.1 ± 7.6 years; 177.2 ± 7.1 cm; 75.8 ± 8.4 kg; 24.1 ± 2.1 kg·m^−2^) volunteered to participate in this pilot study. Subjects were suitable for eligibility if: (i) >18 years old; (ii) >2 years of RT experience; (iii) were committed to adhere to their respective prescribed training protocol with no other physical activity performed nor extra dietary supplements consumed during the 8-week study period. Taking any performance and image-enhancing drugs in the 2-year period prior to the study were considered as exclusion criteria. The participants were informed about the experimental protocol and the potential associated risks. The research protocol was approved by the Ethics Committee of the EADE-University of Wales Trinity Saint David (code: EADECAFYD-2019) and developed in accordance to the ethical guidelines of the Declaration of Helsinki [41].

### 2.3. Intervention Procedures

Experimental procedures were conducted as previous studies carried out by our research group [26,42]. Both experimental groups (CS-RT+CrM and CS-RT) trained to volitional failure twice a week with 72 h of rest between sessions. The exercise intervention program was performed within the infrastructure of the fitness and strength conditioning center ‘Physical Training’ (Málaga, Spain) while measurements were taken in the Human Kinetics and Body Composition Laboratory at the University of Málaga.

#### 2.3.1. Anthropometry

All anthropometric data were collected during the first visit to the laboratory during the familiarization weeks. Body mass was measured with a digital scale to the nearest 50 g (Tanita RD-545, Tokyo, Japan). A fixed stadiometer was used to measure the stature (SECA 220, Hamburg, Germany).

#### 2.3.2. Exercise Protocol

All participants were familiarized with advanced resistance training strategies. The CS-RT+CrM and CS-RT groups performed the same training protocol during the eight weeks of the study while subjects of the control group were asked to maintain their habitual training regimes throughout the study (Table 1). Three exercises were performed in the following order: squat (3 sets), deadlift (3 sets), and leg press (2 sets). An unsupervised standardized training program for upper limbs was prescribed in the experimental groups. Training sessions were monitored by strength and conditioning specialists, adjusting the loads whenever necessary.

#### 2.3.3. Dietary Intervention

All participants were prescribed to consume ∼39 kcal·kg^−1^ FFM per day in order to optimize changes in body composition [43]. In the CS-RT+CrM and CS-RT groups, participants were instructed to consume the following macronutrient distribution: 5.0 g·kg^−1^ FFM·day^−1^ of carbohydrates, 2.5 g·kg^−1^ FFM·day^−1^ of protein, and 1.0 g·kg^−1^ FFM·day^−1^ of fat [44]. Similar foods were recommended for the diets of participants in the CS-RT+CrM and CS-RT groups while subjects in the control group maintained their habitual diet. The CS-RT+CrM group followed an eight-week CrM supplementation protocol of 0.1 g·kg^−1^·day^−^^1^ (Creatine-Red Gold Series, MTX Corporation, Irún, Spain). Several studies have shown that this chronic supplementation protocol (with or without loading phase) is safe and effective for improving exercise performance capacity and training adaptations in trained men [12,13]. The individualized amount of CrM was fully dissolved in ≈500 mL of a shake with 0.5 g·kg^−1^ beef protein (Carnicode, MTX Corporation, Irún, Spain) and given to the participants immediately after each training session (in the morning on non-training days). Both CS-RT+CrM and CS-RT groups consumed 0.5 g·kg^−1^ protein post-exercise and, therefore, we equated protein consumption between experimental groups. A sport nutritionist designed and supervised all individualized protocols to optimize dietary adherence and confirm the dietary compliance (total daily energy intake and macronutrient distribution).

### 2.4. Outcomes

Primary and secondary outcomes were measured following laboratory procedures reported in previous articles published by our research group [26,42].

#### 2.4.1. Primary Outcome Measures

Whole and regional body composition were estimated using dual-energy X-ray absorptiometry (DXA). Each subject was scanned by a certified technician, and the distinguished bone and soft tissue, edge detection, and regional demarcations were performed by computer algorithms (software version APEX 3.0, Hologic QDR 4500, Bedford, MA, USA). The lower-limb region included the foot, lower leg, and upper leg and was defined by an inclined line passing just below the pelvis crossing the neck of the femur. For each scan, subjects wore sports clothes and were asked to remove all materials that could attenuate the X-ray beam. This included jewelry items and underwear containing a wire. Calibration of the densitometer was checked daily against the standard calibration block supplied by the manufacturer. To determine intertester reliability, two different observers manually selected the area for each subject (coefficient of variation ranged from 1.0 to 2.0%).

To eliminate the influence of the fat-free adipose tissue (FFAT), we adjusted the DXA measurements based on the model proposed by Heymsfield et al. (2002) [45]. This model describes that 85% of adipose tissue is fat while the remaining 15% is the estimated fat-free component. Eliminating the influence of FFAT on DXA-derived fat-free mass (FFM) has been shown to provide more accurate values to detect changes in body composition [46]. Therefore, to calculate adipose tissue mass we based our method on the one employed by Abe et al. (2018) [47] and adjusted the DXA-derived vales as follows: first, we estimated the adipose tissue as DXA fat mass ÷ 0.85; FFAT was then calculated as adipose tissue × 0.15; finally, DXA FFM was adjusted with the elimination of FFAT.

#### 2.4.2. Secondary Outcome Measures

##### Muscle Strength (Repetition Maximum Test)

The 1 repetition maximum (1RM) for the back squat (SQ) was assessed in a Smith machine (Gervasport, Madrid, Spain) following procedures reported previously [26,42]. Participants reported to the laboratory having abstained from any exercise other than activities of daily living for at least 72 h before the reference test and at least 72 h before post-study testing. All men performed a general warm-up prior to testing, which consisted of 7 to 10 min of light cardiovascular exercise. A specific warm-up set of the given exercise was then provided for 12 to 15 repetitions with approximately 40% of the 1RM perceived by the participants, with a load progression for each exercise of 3 to 6 load increments. The increases in each load were approximately 10% 1RM until reaching a mean propulsive velocity of 0.5 m·s^−1^ [8], followed by increments of 5 to 10 kg until attainment of 1RM. A rest interval of three to five minutes was afforded between each successive attempt. Participants had to reach parallel in the 1RM SQ for the trial to be considered a successful attempt. The protocol followed the recommendations described by McGuigan [48] and the technical execution of the squat according to Caulfield & Berninger [49].

##### Muscle Power (Countermovement Jump Test)

Participants were instructed to avoid vigorous exercise for 72 h before the tests in both the pre- and post-test periods. Prior to testing, all men performed a general warm-up consisting of light stretching and stationary cycling for 10–12 min. The countermovement jump (CMJ) test was performed on a jump mat (Smart Jump; Fusion Sport, Coopers Plains, Australia) as we have reported previously [26]. Participants were instructed to initiate each jump by squatting to 90° of knee flexion while keeping their hands at the waist and trunk erect, emphasizing that the movement should be performed without interruption from beginning to end. A total of 3–5 attempts were permitted for familiarization. Thereafter, two jumps were recorded with a rest interval of 1 min between each trial; the highest value was used for analysis (coefficient of variation of the technician was 4.65%).

### 2.5. Sample Size

Non-probability sampling (convenience sampling) was implemented as it is often a strategy used in pilot studies. This leads to gain insight before a full-fledged research activity takes place [50]. After the call to participate in this study, 24 subjects were suitable for eligibility from the available population (i.e., resistance-trained men attending the fitness and strength conditioning center ‘Physical Training’ located in Malaga, Spain).

### 2.6. Randomization

Subjects were randomly assigned (www.randomizer.org accessed on 3 July 2021) to three groups using a 1:1 allocation ratio design: the CS-RT+CrM group (*n* = 9), the CS-RT group (*n* = 9), and the control group (*n* = 9). Subsequently, familiarization and baseline measurements were performed.

### 2.7. Statistical Analysis

The descriptive statistics are expressed as mean and standard deviation (SD). To determine statistical significance, we examined the 95% CIs for the difference between the mean change scores (Δ = Week 8–Week 0). If the 95% CI excludes zero, the difference will attain significance at the *p* < 0.05 level. Effect size was calculated as unbiased Cohen’s *d* (d_unb_), considering a result of ≤0.2 as a small, 0.5 as a moderate, ≥0.8 as a large effect, and ≥1.30 as a very large effect [51]. Estimation plots were generated to display the repeated measures data across two time points (at baseline and after eight weeks). A difference-in-differences (Diff-in-Diff) analysis was performed to compare changes in the outcome variables between the groups, as we have implemented previously [52]. Complementarily, the Kruskall–Wallis test was used for the pairwise comparisons of the Δ between groups. Statistical analyses were performed using IBM SPSS, version 26 (IBM Corp., Armonk, NY, USA), and the Exploratory Software for Confidence Intervals [53].

## 3. Results

**Figure 2:**
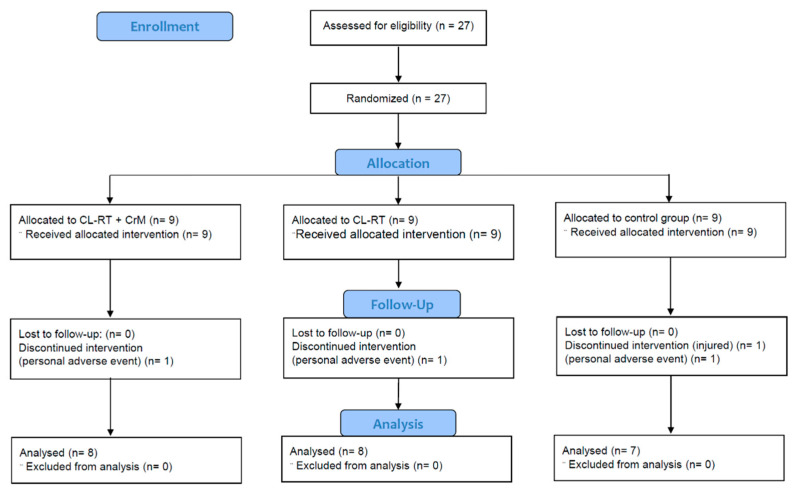
CONSORT flow diagram.

Twenty-three men (26.6 ± 8.1 years; 176.3 ± 6.8 cm; 75.6 ± 8.9 kg; 24.3 ± 2.0 kg·m^−2^) completed the study and were included in the analysis. One participant from the control group discontinued intervention due to injury. Three participants (one per group) were excluded from the analysis because of personal adverse events (Figure 2).

### 3.1. Baseline Data

Analysis of baseline characteristics showed that there were no statistical differences (Kruskal–Wallis test, *p* > 0.05) between the groups for the studied variables (Table 2).

### 3.2. Outcomes

The results of all variables are expressed as Δ ± SD [95% CI]; d_unb_ [95% CI] and presented in Table 3. After eight weeks, there were no significant differences in body mass in any group. Whole-body fat mass and fat percentage had a moderately significant decrease in the groups CS-RT+CrM (−2.18 ± 0.82 (−2.88, −1.49); −0.44 (−0.77, −0.20) and −1.75 ± 1.41 (−2.94, −0.56); −0.47 (−0.93, −0.11), respectively) and CS-RT (−1.75 ± 1.41 (−2.94, −0.56); −0.47 (−0.93, −0.11) and −1.32 ± 1.12 (−2.26, −0.37); −0.36 (−0.64, −0.07), respectively) while moderate and small effects were detected for these variables in the control group. FFM (corrected for FFAT) increased significantly in all groups although a higher effect size was found in the participants of the CS-RT+CrM group (2.95 ± 1.68 (1.54, 4.35); 0.64 (0.24, 1.19)) in comparison to the CS-RT (1.57 ± 1.09 (0.65, 2.48); 0.16 (0.05, 0.30)) and control (0.87 ± 1.91 (−0.89, 2.64); 0.13 (−0.11, 0.41)) groups. A statistically significant and moderate reduction in lower-limb fat mass was observed in the CS-RT+CrM (−0.87 ± 0.44 (−1.24, −0.51); −0.28 (−0.51, −0.11)) and CS-RT (−0.83 ± 0.72 (−1.44, −0.22); −0.35 (−0.71, −0.07)) groups with a small effect for the control group (−0.45 ± 0.50 (−0.92, 0.006); −0.15 (−0.34, 0.002)). Finally, both experimental groups presented significant changes in lower-limb FFM (corrected for lower-limb FFAT), although the participants supplemented with CrM had a higher effect size (0.62 versus 0.18 for the CS-RT+CrM and CS-RT groups, respectively). No significant change was detected in the control group for lower-limb FFM.

**Figure 3:**
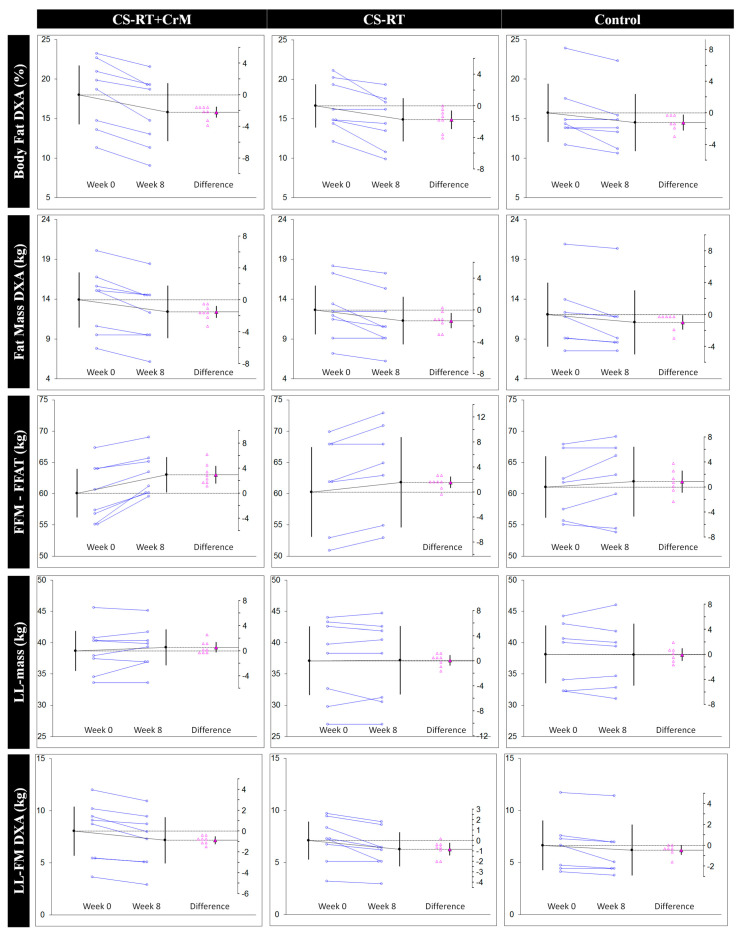

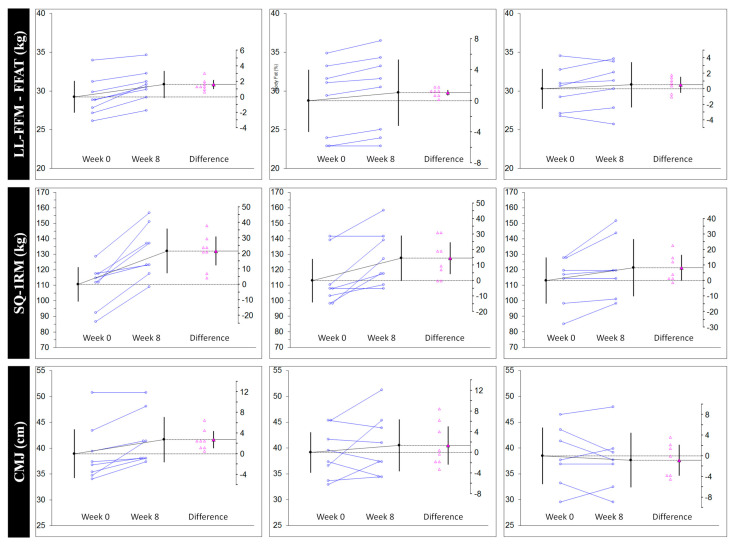
Estimation plots showing pre- and post-intervention values on analyzed variables. Paired data from CS-RT+CrM (left), CS-RT (middle), and control (right) groups are shown as small circles joined by blue lines. The differences between the initial (Week 0) and final (Week 8) means are plotted on a floating difference axis whose zero is aligned with the pre-test mean. The filled pink triangle marks the difference on that axis and the 95% CI on that difference is displayed. The differences are shown as open triangles on the difference axis. FFM and LL-FFM were corrected for fat-free adipose tissue. BMI, body mass index; CI, confidence interval; CMJ, countermovement jump; CrM, creatine monohydrate; CS-RT, cluster-set resistance training; FFAT, fat-free adipose tissue; FFM, fat-free mass; FM, fat mass; LL-FFAT; lower-limb fat-free adipose tissue; LL-FFM; lower-limb fat-free mass; LL-FM; lower-limb fat mass; LL-mass, lower-limb mass; SQ-1RM, 1 repetition maximum for the back squat.

In regard to lower-limb strength, a statistically significant increase in SQ-1RM was found in the CS-RT+CrM and CS-RT groups with no significant changes in the control participants (8.31 ± 9.02 (−0.03, 16.66); 0.40 (−0.001, 0.89)). However, men supplemented with CrM showed a large effect size (21.53 ± 11.19 (12.17, 30.89); 1.23 (0.50, 2.25)) in comparison to the moderate effect seen in the CS-RT group (14.50 ± 12.27 (4.24, 24.75); 0.75 (0.17, 1.48)). Only participants of the CS-RT+CrM group showed a moderate statistically significant improvement in lower-limb muscle power measured as CMJ (2.72 ± 1.99 (1.06, 4.39); 0.44 (0.13, 0.85)) while no significant changes were observed in the CS-RT (1.36 ± 4.42 (−2.34, 5.06); 0.22 (−0.33, 0.82)) and control (−0.84 ± 3.23 (−3.82, 2.14); −0.12 (−0.55, 0.28)) groups. Figure 3 shows paired results between initial and final measurements.

**Figure 4:**
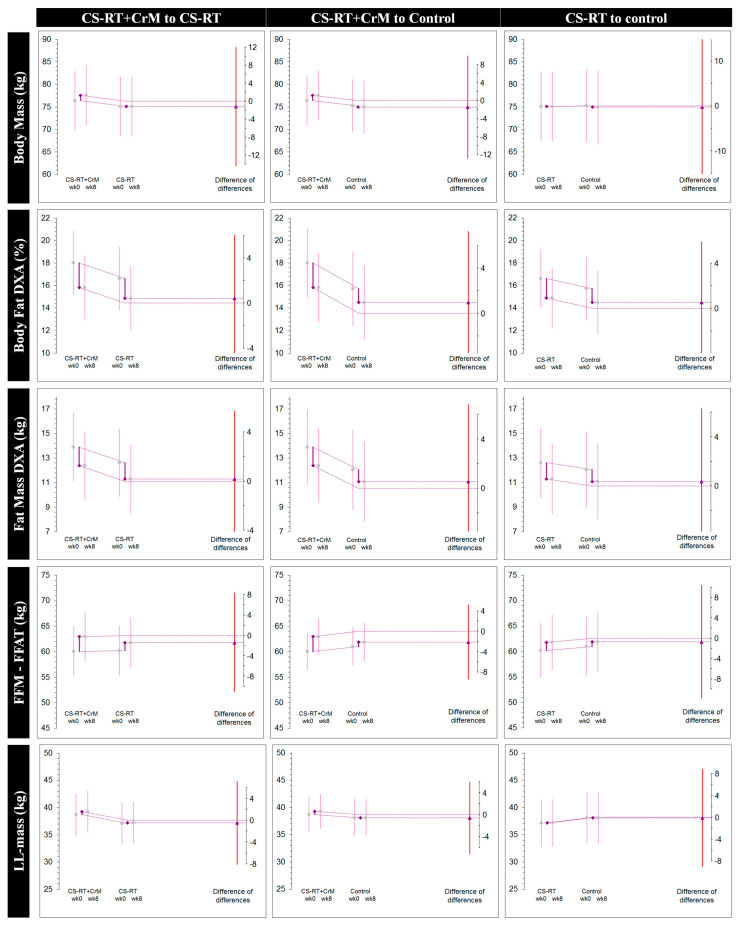

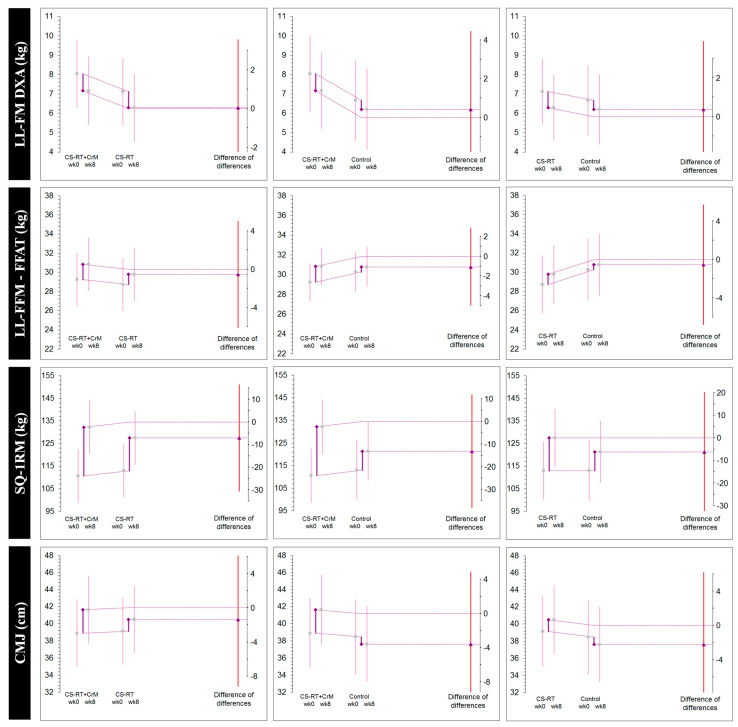
Difference-in-difference estimation plots for all variables. This graphic shows the difference (Δ = Week 8–Week 0) of the differences, which is the calculation of the group means: CS-RT+CrM (Δ_1_), CS-RT (Δ_2_), and control (Δ_3_) groups on lower-limb body composition and strength-related variables. The effect chosen for examination is displayed as the triangle, with its 95% CI, against a floating different axis.

The independent between-group Diff-in-Diff analysis (Table 4) showed no statistical differences. This was confirmed by performing a Kruskall–Wallis test for the pairwise comparisons of the Δ between groups (all *p* > 0.05). Figure 4 shows the change in the outcome variables in the experimental groups compared to the change in the outcome in the control group.

## 4. Discussion

The effects of CrM supplementation on body composition and RT performance have been ratified over 30 years of clinical research in different populations [12,14,15,16]. However, this study is expected to be the first literature contribution on the combination of CrM supplementation and a CS-RT program. An 8-week lower-limb CS-RT program (four clusters of 3RM per set with 20 s of intra-set rest period and 180 s of inter-set rest) was carried out twice per week with 72 h of recovery between sessions. We measured the effects of a CrM supplementation protocol (0.1 g·kg^−1^·day^−1^) during this CS-RT program on lower-limb body composition and strength/power-related variables in resistance-trained men. Since CS-RT takes advantage of the intra-set rest periods to allow PCr resynthesis, which can evoke potentially greater benefits than traditional RT [32,33], we hypothesized that CrM would have a superior impact on the exercise adaptations to this training strategy in resistance-trained men that aim to optimize body composition and muscle strength.

Significant improvements were observed after the CS-RT program in the participants supplemented with or without CrM on the main studied variables (i.e., whole-body fat percentage, fat mass, whole-body FFM, lower-limb fat mass, and lower-limb FFM). However, higher clinical meaningfulness (larger effect sizes) was obtained in the CS-RT+CrM group regarding whole-body FFM (0.64 versus 0.16), lower-limb FFM (0.62 versus 0.18), and SQ-1RM (1.23 versus 0.75) when compared to the CS-RT group. Interestingly, lower-limb muscle power only improved significantly after CrM supplementation (d_unb_ = 0.44) with no significant changes in the CS-RT nor the control group. Thus, although no significant difference between groups was detected, we partially confirmed our initial hypothesis. In fact, a similar but not identical RT methodology known as drop-set RT has also been shown to be benefited by CrM supplementation [54]; nevertheless, the referred study included untrained aging males, which remarks the novel findings of our research.

It is highly possible that optimization of the CK/PCr system after CrM supplementation allow energy and metabolic buffering (regulation of Pi, H^+^ and Ca^2+^ concentrations), which might result in a higher training volume with the same mechanical output. Some researchers have suggested recently that the CK/PCr system might act as a dynamic biosensor of the cellular chemo-mechanical energy transduction (cellular allostasis) [8]. This is important at the whole-body level if we consider that the altered phenotype of an individual is a result of an allostatic load that is sustained for an appropriate interval of time; hence, the faster the recovery, the sooner the desired alteration in the phenotype [55]. More studies in female and untrained exercisers are needed to confirm the potential optimization of CS-RT adaptations by CrM, especially if the similar effects of traditional RT and CS-RT in postmenopausal and elderly women are taken into account [56].

We evaluated effects on lower-limb body composition and strength due to the marked response in this group of muscles after CrM administration [57] and the potential sports- and health-related benefits that can be derived from CS-RT [58]. Hence, the clinical importance of our findings (moderate and large effect sizes) open an interesting line of research in other several areas. Thus, further research is warranted on a wide range of phenotypes that can be benefited from the combination of CS-RT and CrM supplementation, including age-related loss of lower-limb mass (sarcopenia) [59], age-related loss of lower-limb muscle strength/power (dynapenia) [60], cancer-related impairments of lower-limb neurological function and skeletal muscle mass [61], osteoarthritis-related low skeletal muscle mass in the lower limbs [62], and the potential of CrM to reduce hemodialysis-related sarcopenia and improve quality of life in hemodialysis patients [63,64].

It is noteworthy that we not only used an evidence-based nutritional strategy to optimize increases in lean body mass and strength (high-protein hyperenergetic diet) [43,65,66] but also analyzed DXA body composition data after adjusting for FFAT to accurately estimate the changes in lean tissue [46]. We are aware that as a result of the skewed selection of participants this study is susceptible to bias and other forms of selection errors [67]. However, besides serving as a time-efficient strategy with lower financial expenditures, this small-scale feasibility study allows us to evaluate the practicability of carrying out an intervention in a larger future study [50], in this case on the effects of CrM supplementation during a CS-RT program.

## 5. Conclusions

Resistance-trained men following a high-protein diet and a CS-RT program with or without CrM supplementation improved body composition and strength-related variables in lower limbs after eight weeks, while no changes were detected in participants following an unsupervised nutrition and RT exercise regime. Notwithstanding, the supplementation with CrM promoted greater exercise adaptations considering the higher clinical meaningfulness (larger effect sizes) on whole-body FFM, lower-limb FFM, and SQ-1RM in comparison to the CS-RT and control groups. Although double-blinded clinical trials with a larger sample are needed to confirm these findings, the combination of CrM supplementation and CS-RT seems a practical strategy to optimize training adaptation in advanced exercisers that aim to increase lower-limb FFM and strength. Further research might study the potential health and therapeutic effects of this nutrition and exercise strategy.

## Figures and Tables

**Table 1:** Eight-week cluster-set resistance training protocol.

Group	Exercises	Sets per Exercise	Reps per Set	Clusters per Set	Intra-Set Rest (s)	Inter-Set Rest (s)
CS-RT+CrM	Squat, deadlift, and leg press *	3	12	4 × 3RM	20	180
CS-RT		3	12	4 × 3RM	20	180
Control	Followed their habitual diet and training programs (no recorded)					

Participants were encouraged to train on Monday and Thursday to favor recovery given all reported having lower energy levels and mechanical expenditure during the weekend. * Two sets were performed for the leg press exercise.

**Table 2:** Descriptive information of participants at baseline.

Variable	CS-RT+CrM (*n* = 8) $\bar{x}$ (SD)	95% CI (min, max)	CS-RT (*n* = 8) $\bar{x}$ (SD)	95% CI (min, max)	Control (*n* = 7) $\bar{x}$ (SD)	95% CI (min, max)
Body mass (kg)	76.41 (6.72)	70.79, 82.03	75.09 (11.69)	65.31, 84.86	75.22 (8.83)	67.04, 83.39
Stature (cm)	179.41 (5.07)	175.16, 183.65	173.31 (7.72)	166.85, 179.77	176.45 (7.47)	169.54, 183.36
BMI (kg∙m^−2^)	23.89 (1.71)	22.45, 25.32	25.02 (2.73)	22.73, 27.31	24.25 (2.51)	22.82, 25.68
Body fat_DXA_ (%)	18.00 (4.45)	14.27, 21.73	16.63 (3.27)	13.89, 19.36	15.72 (3.99)	12.02, 19.41
FM_DXA_ (kg)	13.89 (4.15)	10.42, 17.37	12.62 (3.65)	9.56, 15.67	12.04 (4.32)	8.04, 16.05
FFAT_total_ (kg)	2.45 (0.73)	1.84, 3.06	2.22 (0.64)	1.68, 2.76	2.12 (0.76)	1.42, 2.83
FFM_DXA_ (kg)	62.51 (4.69)	58.59, 66.43	62.47 (8.98)	54.95, 69.98	63.17 (5.69)	57.90, 68.44
FFM_DXA_ minus FFAT_total_ (kg)	60.06 (4.63)	56.18, 63.93	60.24 (8.58)	53.06, 67.42	61.04 (5.32)	56.12, 65.96
LL-mass (kg)	38.70 (3.83)	35.49, 41.90	37.10 (6.55)	31.6, 42.58	38.10 (4.97)	33.50, 42.71
LL-FM_DXA_ (kg)	8.03 (2.83)	5.67, 10.40	7.11 (2.17)	5.30, 8.93	6.66 (2.58)	4.27, 9.05
LL-FFAT (kg)	1.41 (0.49)	1.00, 1.83	1.25 (0.38)	0.93, 1.57	1.17 (0.45)	0.75, 1.59
LL-FFM_DXA_ (kg)	30.66 (2.42)	28.63, 32.69	29.98 (5.01)	25.78, 34.18	31.44 (3.01)	28.65, 34.23
LL-FFM minus LL-FFAT (kg)	29.24 (2.45)	27.19, 31.29	28.72 (4.79)	24.71, 32.74	30.27 (2.77)	27.70, 32.84
SQ-1RM (kg)	110.62 (13.36)	99.45, 121.79	113.00 (16.82)	98.93, 127.06	113.00 (16.02)	98.18, 127.81
CMJ (cm)	38.92 (5.64)	34.20, 43.64	39.14 (4.69)	35.21, 43.06	38.47 (5.91)	33.00, 43.95

Data are expressed as mean (standard deviation) and 95% confidence interval. FFM and LL-FFM were corrected for fat-free adipose tissue. BMI, body mass; CI, confidence interval; CMJ, countermovement jump; CrM, creatine monohydrate; CS-RT, cluster-set resistance training; FFAT, fat-free adipose tissue; FFM, fat-free mass; FM, fat mass; LL-FFAT; lower-limb fat-free adipose tissue; LL-FFM; lower-limb fat-free mass; LL-FM; lower-limb fat mass; LL-mass, lower-limb mass; SQ-1RM, 1 repetition maximum for the back squat.

**Table 3:** Pre- and post-intervention data on the main study variables.

Variable	Group	Week 0 $\bar{x}$ (SD)	Week 8 $\bar{x}$(SD)	Δ $\bar{x}$ (SD) [95% CI]	d_unb_ δ [95% CI]
Body mass (kg)	CS-RT+CrM	76.41 (6.72)	77.59 (4.89)	1.18 (2.33) [–0.76, 3.13]	0.17 [–0.09, 0.48]
	CS-RT	75.09 (11.69)	75.11 (11.34)	0.01 (1.17) [–0.96, 1.00]	0.00 [–0.06, 0.07]
	Control	75.22 (8.83)	74.96 (9.52)	–0.25 (2.16) [–2.25, 1.74]	–0.02 [–0.20, 0.14]
Body fat_DXA_ (%)	CS-RT+CrM	18.00 (4.45)	15.81 (4.37)	–2.18 (0.82) [–2.88, –1.49] *	–0.44 [–0.77, –0.20]
	CS-RT	16.63 (3.27)	14.87 (3.28)	–1.75 (1.41) [–2.94, –0.56] *	–0.47 [–0.93, –0.11]
	Control	15.72 (3.99)	14.49 (3.91)	–1.22 (1.08) [–2.23, –0.22] *	–0.27 [–0.57, –0.03]
FM_DXA_ (kg)	CS-RT+CrM	13.89 (4.15)	12.39 (3.92)	–1.50 (0.88) [–2.24, –0.76] *	–0.33 [–0.61, –0.12]
	CS-RT	12.62 (3.65)	11.30 (3.56)	–1.32 (1.12) [–2.26, –0.37] *	–0.36 [–0.64, –0.07]
	Control	12.04 (4.32)	11.08 (4.35)	–0.96 (0.97) [–1.86, –0.06] *	–0.19 [–0.42, –0.01]
FFM_DXA_ minus FFAT_total_ (kg)	CS-RT+CrM	60.06 (4.63)	63.01 (3.35)	2.95 (1.68) [1.54, 4.35] *	0.64 [0.24, 1.19]
	CS-RT	60.24 (8.58)	61.81 (8.62)	1.57 (1.09) [0.65, 2.48] *	0.16 [0.05, 0.30]
	Control	61.04 (5.32)	61.92 (6.04)	0.87 (1.91) [–0.89, 2.64]	0.13 [–0.11, 0.41]
LL-mass (kg)	CS-RT+CrM	38.70 (3.83)	39.26 (3.44)	0.55 (0.99) [–0.27, 1.38]	0.13 [–0.05, 0.35]
	CS-RT	37.10 (6.55)	37.17 (6.53)	0.07 (1.00) [–0.76, 0.91]	0.01 [–0.09, 0.11]
	Control	38.10 (4.97)	38.09 (5.34)	–0.01 (1.08) [–1.01, 0.99]	–0.002 [–0.15, 0.15]
LL-FM_DXA_ (kg)	CS-RT+CrM	8.03 (2.83)	7.16 (2.65)	–0.87 (0.44) [–1.24, –0.51] *	–0.28 [–0.51, –0.11]
	CS-RT	7.11 (2.17)	6.28 (1.96)	–0.83 (0.72) [–1.44, –0.22] *	–0.35 [–0.71, –0.07]
	Control	6.66 (2.58)	6.20 (2.64)	–0.45 (0.50) [–0.92, 0.006] *	–0.15 [–0.34, 0.002]
LL-FFM minus LL-FFAT (kg)	CS-RT+CrM	29.24 (2.45)	30.83 (2.09)	1.59 (0.70) [1.00, 2.18] *	0.62 [0.27, 1.11]
	CS-RT	28.72 (4.79)	29.78 (5.10)	1.05 (0.43) [0.69, 1.41] *	0.18 [0.08, 0.33]
	Control	30.27 (2.77)	30.79 (3.15)	0.52 (1.12) [–0.51, 1.56]	0.15 [–0.12, 0.46]
SQ-1RM (kg)	CS-RT+CrM	110.62 (13.36)	132.16 (17.27)	21.53 (11.19) [12.17, 30.89] *	1.23 [0.50, 2.25]
	CS-RT	113.00 (16.82)	127.50 (17.48)	14.50 (12.27) [4.24, 24.75] *	0.75 [0.17, 1.48]
	Control	113.00 (16.02)	121.31 (19.87)	8.31 (9.02) [–0.03, 16.66]	0.40 [–0.001, 0.89]
CMJ (cm)	CS-RT+CrM	38.92 (5.64)	41.65 (5.23)	2.72 (1.99) [1.06, 4.39] *	0.44 [0.13, 0.85]
	CS-RT	39.14 (4.69)	40.50 (6.01)	1.36 (4.42) [–2.34, 5.06]	0.22 [–0.33, 0.82]
	Control	38.47 (5.91)	37.63 (5.71)	–0.84 (3.23) [–3.82, 2.14]	–0.12 [–0.55, 0.28]

Data is presented as mean ($\bar{x}$) and standard deviation (SD). FFM and LL-FFM were corrected for fat-free adipose tissue. BMI, body mass index; CI, confidence interval; CMJ, countermovement jump; CrM, creatine monohydrate; CS-RT, cluster-set resistance training; FFAT, fat-free adipose tissue; FFM, fat-free mass; FM, fat mass; LL-FFAT; lower-limb fat-free adipose tissue; LL-FFM; lower-limb fat-free mass; LL-FM; lower-limb fat mass; LL-mass, lower-limb mass; SQ-1RM, 1 repetition maximum for the back squat. * Statistically significant change (*p* < 0.05).

**Table 4:** Difference of differences between the studied groups.

	DID for CS-RT+CrM (Δ_1_) and CS-RT (Δ_2_)				DID for CS-RT+CrM (Δ_1_) and Control (Δ_3_)				DID for CS-RT (Δ_2_) and Control (Δ_3_)			
Variable	Mean (Δ_2_–Δ_1_)	DID	95% CI	*p*	Mean (Δ_3_–Δ_1_)	DID	95% CI	*p*	Mean (Δ_3_–Δ_2_)	DID	95% CI	*p*
Body mass (kg)	0.01–1.18	−1.16	−14.41, 12.08	0.858	−0.25–1.18	−1.44	−12.86, 9.97	0.797	−0.25–0.01	−0.27	−16.09, 15.53	0.972
Body fat_DXA_ (%)	−1.75–−2.18	0.43	−5.20, 6.07	0.876	−1.22–−2.18	0.96	−5.37, 7.29	0.758	−1.22–−1.75	0.52	−4.90, 5.95	0.843
FM_DXA_ (kg)	−1.32–−1.50	0.18	−5.36, 5.73	0.947	−0.96–−1.50	0.53	−5.75, 6.83	0.862	−0.96–−1.32	0.35	−5.60, 6.31	0.903
FFM_DXA_ minus FFAT total (kg)	1.57–2.95	−1.38	−11.12, 8.34	0.774	0.87–2.95	−2.07	−9.41, 5.25	0.566	0.87–1.57	−0.69	−11.84, 10.44	0.899
LL-mass (kg)	0.07–0.55	−0.48	−8.15, 7.18	0.898	−0.01–0.55	−0.57	−7.21, 6.06	0.861	−0.01–0.07	−0.087	−9.03, 8.86	0.984
LL-FM_DXA_ (kg)	−0.83–−0.87	0.04	−3.47 3.56	0.980	−0.45–−0.87	0.41	−3.62, 4.46	0.833	−0.45–−0.83	0.37	−3.14, 3.89	0.828
LL-FFM minus LL-FFAT (kg)	1.05–1.59	−0.53	−6.12, 5.04	0.845	0.52–−1.59	−1.06	−5.01, 2.88	0.583	0.52–1.05	−0.53	−6.78, 5.72	0.863
SQ-1RM (kg)	14.50–21.53	−7.03	−30.68, 16.60	0.547	8.31–21.53	−13.22	−38.34, 11.89	0.289	8.31–14.50	−6.18	−32.63, 20.25	0.635
CMJ (cm)	1.36–2.72	−1.36	−9.21, 6.48	0.724	−0.84–2.72	−3.56	−12.02, 4.88	0.394	−0.84–1.36	−2.203	−10.62, 6.21	0.595

DID: Difference of differences. The *p* value is two-tailed with statistical significance when <0.05.

## Data Availability

The data that support the findings of this study are available upon request from the corresponding author.
